# The Ultimate Micro-Exon: A Single Nucleotide Exon Is Required to Assemble Cytochrome P450 CYP621A Orthologs from *Fusarium* Species

**DOI:** 10.3390/ijms27041979

**Published:** 2026-02-19

**Authors:** David R. Nelson, Khajamohiddin Syed

**Affiliations:** 1 Department of Microbiology, Immunology and Biochemistry, University of Tennessee Health Science Center, Memphis, TN 38163, USA; 2 Department of Biochemistry and Microbiology, Faculty of Science, Agriculture and Engineering, University of Zululand, Empangeni 3886, South Africa

**Keywords:** Cytochrome P450 monooxygenases, micro-exon, exon, *Fusarium* species, gene annotation, splicing

## Abstract

Cytochrome P450 monooxygenases (CYPs/P450s) play a key role in organisms’ primary and secondary metabolism in species across all domains of life. Accurate annotation of P450 genes is crucial for identifying their functions, evolution, and, consequently, their biotechnological potential. In this study, we report the identification of an unprecedented one-nucleotide exon required for the correct assembly of *CYP621A* P450 genes from multiple *Fusarium* species. Through comparative genomic analysis of 20 orthologous *CYP621A* genes, supported by an intronless *CYP621B1* gene from *Aspergillus clavatus*, we demonstrate that omission of this single-nucleotide exon disrupts exon phase compatibility and prevents reconstruction of a full-length, functional P450 protein. The micro-exon encodes the central nucleotide of the glycine codon in the highly conserved PKG motif, which is essential for maintaining the structural integrity between the EXXR and PERF motifs, a characteristic of P450 enzymes. Importantly, transcriptomic evidence from sequence read archive (SRA) data confirms accurate splicing of this one-nucleotide exon in *Fusarium solani* and *F. acuminatum* under multiple growth conditions. This work presents the second example of the smallest exon reported to date for a gene, and the first for a P450 gene or a fungal gene. The study’s findings have broad implications for genome annotation pipelines, underscoring the need for careful manual curation and improved algorithms to detect ultra-small exons in functionally constrained regions of eukaryotic genes.

## 1. Introduction

Cytochrome P450 monooxygenases (CYPs/P450s) are heme-containing proteins ubiquitously found across all domains of life, including viruses [1]. P450s catalyze diverse reactions with stereo- and regio-selectivity [2,3,4,5]. P450s play an important role in primary and secondary metabolism, where they are involved in the biosynthesis or degradation of endogenous compounds and in the synthesis of secondary metabolites valuable to humans [6,7,8,9]. In fungi, P450 enzymes have dual functions: they serve as drug targets (CYP51 and CYP53 families) against pathogenic fungi [10,11,12] and play important roles in primary and secondary metabolism [13], enabling adaptation to diverse ecological niches [14]. Given their enormous importance, accurate identification and annotation of P450 genes in fungal genomes are essential for both basic and applied research.

In this current genomic era, where genome sequencing has now become a daily routine, including sequencing of many fungal genomes as part of the Joint Genome Institute’s (JGI) MycoCosm project [15], this has led to the widespread use of computational programs to predict exon–intron structures and thus identify genes (annotation) in a genome sequence. Despite substantial improvements in automated pipelines, they remain challenged by atypical gene features, such as non-canonical splice sites, long introns, and, in particular, very small exons, commonly known as “micro-exons”. Because of their small size, micro-exons are frequently missed or misassembled, leading to frameshifts. Specific algorithms have been developed to identify micro-exons [16] and newer methods for efficiently and accurately mapping cDNAs to genomic sequences have addressed this problem. For example, the algorithm Spaln was able to identify all exons in a test dataset [17]. Exons in the 2–5 base pair size range have been found in *Arabidopsis*, *Drosophila*, and humans [16]. Small exons coding for 2–10 amino acids are also observed in fungi [18]. Interestingly, only a single study has reported a single-nucleotide exon in the *Anaphase Promoting Complex subunit 11* (*APC11*) gene of *Arabidopsis thaliana* [19]. These micro-exon coding elements are often embedded within conserved protein motifs, where any splicing change can compromise protein function. Thus, strong selective pressure exists to preserve both exon length and amino acid composition in these regions.

Despite having very low amino acid sequence identity between distant family members, the P450 three-dimensional structural fold is highly conserved, indicating the presence of P450 family characteristic motifs [20,21]. These motifs include a heme-binding signature motif in the heme-binding domain (FXXGXRXCXG/CXG, where X represents any amino acid) and the EXXR motif in the K-helix, the oxygen-binding motif in the I-helix, and the PERF motif located upstream of the heme-binding region [20,21]. Between the EXXR and PEFR motifs lies a short PKG motif/sequence, which is found in many P450s. Given its position, one can predict that it plays a role in maintaining local structural integrity and facilitating the proper spatial organization of adjacent helices, both of which are critical for P450 fold stability.

In this study, we report a single-nucleotide exon that is required to assemble the PKG motif in 20 *CYP621A* subfamily members from *Fusarium* species and provide transcriptomic evidence confirming the accurate splicing of this ultra-small exon in vivo.

## 2. Results

**Figure 1:**
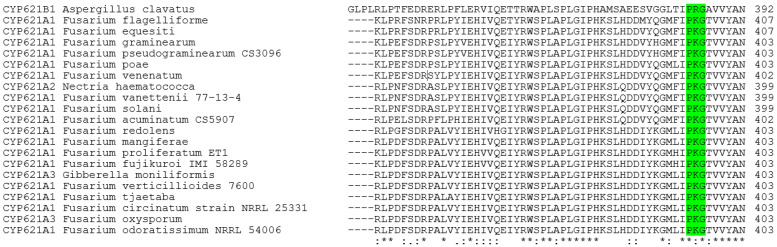
Multiple protein sequence alignment of twenty-one members of the CYP621 family, highlighting the PKG motif region. A full-length alignment of these members is presented as Figure S1.

**Figure 2:**
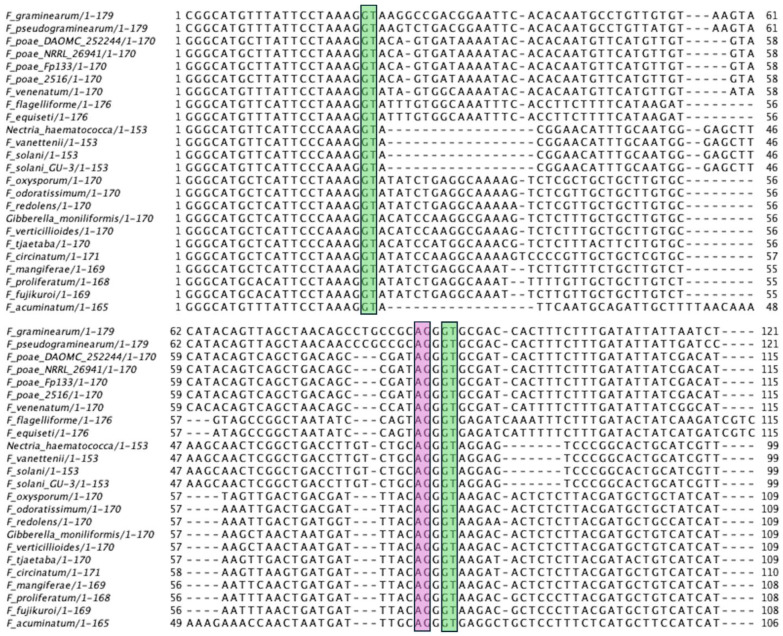

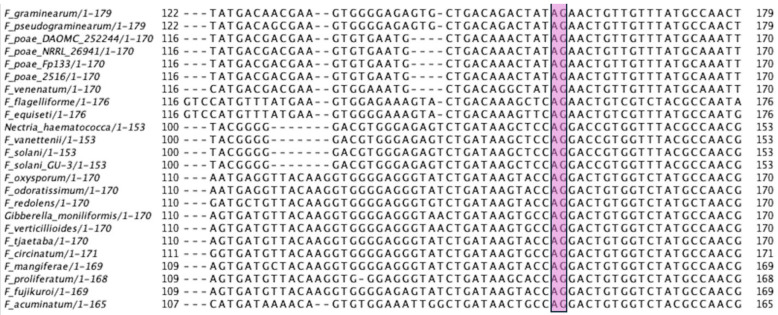
Multiple sequence alignment of the intronic regions surrounding the one-nucleotide exon of twenty-four *CYP621A* subfamily members. The GT (green) and AG (purple) boundaries of the introns are included with 20 nucleotides upstream and downstream that extend into the adjacent exons. The single-nucleotide exon is highlighted with the AG and GT boundaries included. *CYP621B1* is not included because it lacks introns. Information on these P450s is presented in Table S1.

The genes containing a single nucleotide exon are 20 ortholog P450s from *Fusarium oxysporum*, *F. verticillioides* (teleomorph: *Gibberella moniliformis*), *F. graminearum* (teleomorph: *Gibberella zeae*), *F. pseudograminearum*, *F. virguliforme*, *F. circinatum*, *F. equiseti*, *F. solani* (teleomorph: *Haematonectria haematococca*), *Nectria haematococca*, *F. flagelliforme*, *F. Poae*, *F. venenatum*, *F. vanettenii* 77-13-4, *F. acuminatum* CS5907, *F. redolens*, *F. mangiferae*, *F. proliferatum* ET1, *F. fujikuroi* IMI58289, *F. tjaetaba*, and *F. odoratissimum* NRRL54006. The intronless gene CYP621B1 from *A. clavatus* is also included in the study. Detailed information on these P450s is presented in Table S1. These CYP621A subfamily members from *Fusarium* species have several recognizable P450 characteristic motifs, including the heme signature sequence, an invariant EXXR motif, the oxygen-binding region in the I-helix, and a PERF motif just upstream of the heme signature [22]. Between the EXXR and PERF motifs, there is a shorter conserved sequence called the PKG motif, which was also found in these P450s (Figure 1), where a single nucleotide exon codes for the central nucleotide of the “G” codon in the PKG motif (Figure 2).

It has been reported that the median intron size in *F. graminearum* is 56 nucleotides [23]. A multiple sequence alignment of the *F. graminearum* intronic region reveals the presence of a 139-nucleotide intron (Figure 2), more than twice the median intron length. This by itself suggests that a micro-exon may be present. A candidate single-nucleotide exon aligns in all 24 sequences in Figure 2 and is boxed with the AG and GT boundaries. The Eukaryotic Splice Database (http://66.170.16.154/EuSplice/, accessed on 20 January 2026) provides tables of splice-site base frequencies for 21 species, including *Aspergillus fumigatus*, the closest fungus to those included here. The donor splice site (GT) has the consensus G (79.90%), G (99.55%), T (98.83%), A (59.63%), and A (69.49%). The first three positions match exactly for the single nucleotide exon, and the last two positions match partially. The acceptor splice site (AG) has the consensus C (65.43%), A (99.64%), G (99.54%), and G (48.85%). The single-nucleotide exon matches this pattern exactly. More distant positions do not show as strong a preference.

Additional support is provided by a gene in the same *CYP621* family that lacks introns (see Figure 1 and Figure S1). The *A. clavatus* CYP621B1 P450 (top sequence in Figure 1) is used as an intronless length marker, similar to an mRNA sequence, to verify that a one-nucleotide exon is required to assemble these genes correctly.

**Figure 3:**
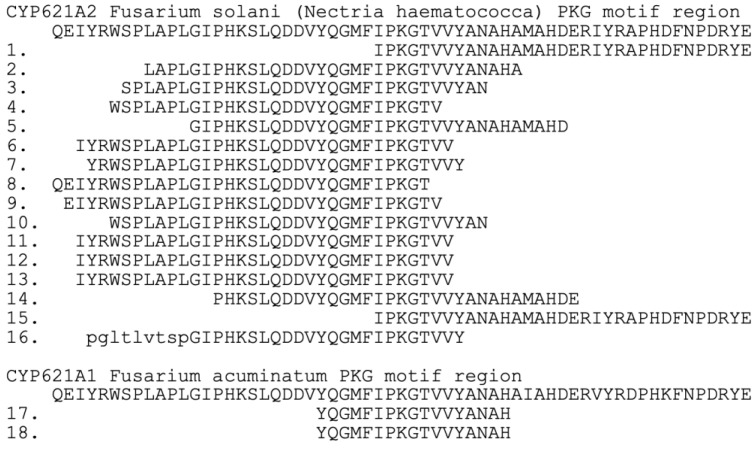
Transcriptome evidence in support of the one-nucleotide exon of CYP621A sequences. Note that sequence 16 has the translated intron sequence pgltlvtsp in lowercase. See Materials and Methods for detailed SRA accessions.

Transcriptomes provide corroborating evidence for this one-nucleotide exon. The *CYP621A* genes seem to be expressed only rarely. Still, two species were found in the Sequence Read Archive (SRA) database with sequences spanning the one-nucleotide exons, verifying the prediction of this unusual joint. Figure 3 presents the 18 SRA reads aligned to the *F. solani* and *F. acuminatum* proteins. The *F. solani* reads were taken from three different growth conditions: SRX1810231 mycelium exposed for 48 h to dimethyl-sulfoxide, SRX1810230 mycelium exposed for 48 h to posaconazole (an anti-fungal drug), SRX1810229 mycelium exposed for 48 h to amphotericin B (an anti-fungal drug). The *F. acuminatum* reads were from mycelium.

## 3. Discussion

During manual annotation of P450 genes from filamentous fungal genomes, the 24 genes described herein were found to have a problem in gene assembly (Table S1). A phase-one boundary at the end of the PKG motif could not be joined to the next detectable downstream exon, which had a phase-two boundary. The nucleotide sequence of the upstream and downstream exons aligned with an intronless gene from the same CYP621 family, with only one base missing at the joint. The solution was to infer a one-nucleotide exon. All 24 of these genes had an identical candidate exon (AGGGT) and conserved intron splice-site sequences. These one-nucleotide exons were first observed in 2009 during the annotation of four genomes: *F. oxysporum*, *F. verticillioides*, *F. graminearum*, and *F. solani*, but no transcriptomic evidence supporting them was available at that time.

The distance between the PKG motif and the PERF motif is 24 amino acids in plants, animals, fungi, and protists, with almost no variation tolerated [24]. The presence of a one-nucleotide exon in these 24 genes preserves the PKG motif, maintains the 24-amino-acid length between the PGK and PERF motifs, and enables proper assembly of a full-length P450.

This observation places the lower limit of exon size at one base, as observed in *A. thaliana* [19]. How can such a small exon evolve? One possible scenario involves the loss of one base in an exon near a GT or AG boundary. If the gene is sufficiently important for the organism, selective pressure would favor restoration of the proper reading frame by adding a base back into one of the exons near the joint. However, if the intron boundary is in a conserved motif, adding a base back randomly near the original site of loss will change the amino acid sequence and damage the motif. In this case, a cryptic exon AGGGT in the intron sequence may be adopted to restore the reading frame. This type of rescue may be more likely if the middle base is a G because G is part of the splice-site consensus for both donor and acceptor splice sites, especially the donor GT site, which has a ~80% G frequency at this position. Another small eight-nucleotide micro-exon gg/cac/gag occurs in a highly conserved P450 I-helix motif AGHETT in the white rot fungus *Phanerochaete chrysosporium* (CYP5141A1, AADS01000627.1, 15226-15233). This short exon begins and ends with G (see http://drnelson.uthsc.edu/Phanerochaete.P450s.htm, accessed on 20 January 2026 for more examples of micro-exons in *Phanerochaete chrysosporium.* There are multiple examples of micro-exons in the I-helix motif). These types of micro-exons may be favored when introns occur within conserved motifs to preserve the motif’s sequence. 

The function of the CYP621A genes is not known. Analysis of upstream and downstream genes/proteins revealed that these *CYP621*s are located near genes/proteins involved in riboflavin biosynthesis. Table S2 has a map of the 4-5 flanking genes for 23 CYP621 genes. There are conserved genes in the immediate neighborhood. Searches using the fungal version of Antismash and the JGI clusters did not find *CYP621*s as part of any gene clusters. Individual searches did uncover a possible common feature. Several genes are most similar to genes involved in riboflavin biosynthesis (Table S2). The adjacent gene, as shown on the left in Table S2, is ribokinase. Ribokinase phosphorylates ribose to make D-ribose-phosphate, which is isomerized to ribulose-5-phosphate (a precursor of riboflavin). Adjacent on the right side is a purine-cytosine permease. Riboflavin is made from GTP, so a purine permease may boost the availability of GTP for synthesis. Another flanking gene is most likely riboflavin biosynthesis protein Rib7. A fourth flanking gene is identified as bifunctional deaminase reductase IPR002734, another riboflavin biosynthesis gene. There is an oxoglutarate iron dependent dioxygenase CUE domain. These genes often act as hydroxylases, as do the cytochrome P450s. Sometimes, P450s and oxoglutarate iron-dependent dioxygenases work in the same pathway, both making hydroxylations, as in gibberellin synthesis. One possible role for CYP621As is the formation of a hydroxylated derivative of riboflavin. The true biological function of these CYP621A enzymes remains to be elucidated.

Given the intron–exon boundary system, life has found a way to take it to the extreme of a single-nucleotide exon. Such exons may explain regions where gene assembly has proven difficult due to out-of-phase intron boundaries.

## 4. Materials and Methods

### 4.1. Information on P450s Used in the Study and Their Annotation

Detailed information on the species, strain, accession numbers, gene IDs, and full protein sequences used in this study is presented in Table S1. However, all transcripts (column D Table S1) built from the gene models in Gene (column E Table S1) are wrong at the PKG motif, except XM_046133393.1 *Fusarium flagelliforme*. See the notes about model errors in column G, Table S1. XM_046133393.1 is correct at the PKG boundary, but the gene model B0J16DRAFT_413818 does not include the one-nucleotide exon, so there is a discrepancy between the transcript XM_046133393.1 and the gene model 70162482. The gene model may not be able to show a one-nucleotide exon. *CYP621B1 A. clavatus* NRRL 1 XM_001274332, locus_tag ACLA_013420 is a correct assembly because there are no introns in the genomic sequence NW_001517104 complement (1359061.1360557). Manual curation and correct annotation of these P450s, along with their multiple alignment highlighting the PKG region, are presented in column K of Table S1 and in Figure S2, respectively.

### 4.2. Information on Sequence Read Archive (SRA) Reads for P450s

The Uniform Resource Locator (URL) for the SRA location of the reads is given. To recover a sequence, go to the URL, click on the run number, and search under reads for the spot number (example for seq 1. run number SRR3609449, under reads, filter search for 10974918). Note that sequence 16 has the translated intron sequence pgltlvtsp in lowercase.

https://www.ncbi.nlm.nih.gov/sra/?term=fusarium+solani+SRX1810231 (accessed on 20 January 2026)

1 = SRX1810231 gnl|SRA|SRR3609449.10974918.1Length: 100

2 = SRX1810231 gnl|SRA|SRR3609449.10974918.2Length: 100

3 = SRX1810231 gnl|SRA|SRR3609447.33844133.2Length: 100

4 = SRX1810231 gnl|SRA|SRR3609447.25291074.2Length: 100

https://www.ncbi.nlm.nih.gov/sra/?term=fusarium+solani+SRX1810230 (accessed on 20 January 2026)

5 = SRX1810230 gnl|SRA|SRR3609444.8194221.1Length: 100

6 = SRX1810230 gnl|SRA|SRR3609444.17343568.2Length: 100

7 = SRX1810230 gnl|SRA|SRR3609445.7850576.1Length: 100

8 = SRX1810230 gnl|SRA|SRR3609445.28658232.2Length: 100

9 = SRX1810230 gnl|SRA|SRR3609445.6764897.2Length: 100

10 = SRX1810230 gnl|SRA|SRR3609445.434239.2Length: 100

11 = SRX1810230 gnl|SRA|SRR3609445.23302647.2Length: 100

12 = SRX1810230 gnl|SRA|SRR3609446.26312239.1Length: 100

13 = SRX1810230 gnl|SRA|SRR3609446.12254076.2Length: 100

https://www.ncbi.nlm.nih.gov/sra/?term=fusarium+solani+SRX1810229 (accessed on 20 January 2026)

14 = SRX1810229 gnl|SRA|SRR3609442.9370392.1Length: 100

15 = SRX1810229 gnl|SRA|SRR3609443.3469891.1Length: 100

16 = SRX1810229 gnl|SRA|SRR3609443.3469891.2Length: 100

https://www.ncbi.nlm.nih.gov/sra/?term=fusarium+acuminatum+SRX5010961 (accessed on 20 January 2026)

17 = SRX5010961 gnl|SRA|SRR8191394.7626861.1Length: 51

18 = SRX5010961 gnl|SRA|SRR8191394.7623381.1.1Length: 51

Transcriptome evidence of *CYP621A2 N. haematococca*/*F. solani* under different growth conditions are presented in Figure S3.

### 4.3. Multiple Sequence Alignment

The Clustal W multiple sequence alignment program [25] was used to align the P450 protein and intronic sequences with default parameters.

## 5. Conclusions

This study reports the discovery and validation of a one-nucleotide exon required for the correct assembly of *CYP621A* P450 genes in *Fusarium* species. This represents the smallest exon described to date in fungi and demonstrates an evolutionary solution to preserve conserved motifs and exon-phase integrity. Our study results underscore the limitations of current automated gene-prediction methods and demonstrate the value of manual curation, supported by comparative genomics and transcriptomic evidence, for identifying single-nucleotide exons in fungal P450 genes. Recognition of such ultra-small exons will be important for improving genome annotations and for accurately interpreting gene structure in functionally constrained regions of eukaryotic genomes.

## Data Availability

Data is contained within the article or Supplementary Material.

## Supplementary Materials

The following supporting information can be downloaded at https://www.mdpi.com/article/10.3390/ijms27041979/s1.
