# Supplementary material for: The Ultimate Micro-Exon: A Single Nucleotide Exon Is Required to Assemble Cytochrome P450 CYP621A Orthologs from Fusarium Species
Source: Int J Mol Sci. 2026 Feb 19;27(4):1979. doi: 10.3390/ijms27041979 (PMC12940212; doi:10.3390/ijms27041979)
Supplement: Supplementary file 1 [file ijms-27-01979-s001.zip › Figure S1.pdf]

Figure S1. Multiple protein sequence alignment of CYP621 family members. The PKG motif is highlighted.

```
CYP621B1_Aspergillus_clavatus                       ---------MESLILLVLIILPASVLIYKRLTPRNLPPSPPGDFFLGHLRRIPSSHAEYQ 51
CYP621A1_Fusarium_flagelliforme                     MIEQFSSAYMPGLALSVV-ALLLFWAY-HPQPTLPLPPGPPSEFLLGHTRVIPQENAAKV 58
CYP621A1_Fusarium_equesiti                          MIEQFSLTYMPGLAFSVV-VLLLFWAY-HLQPILPLPPGPPSEFLLGHTRVIPQENAAKV 58
CYP621A1_Fusarium_graminearum                       MIELYSFAHMLGPALVFT-SLLIFWAHTHFQPTLPLPPGPPSEFLLGHTRVIPKENAAEV 59
CYP621A1_Fusarium_pseudograminearum_CS3096          MIELYSFAHMFGPVLVLT-SLLIFWAHRHFQPTLPLPPGPPSEFLLGHTRVIPKENAAKV 59
CYP621A1_Fusarium_poae                              MIEHYSFAHILGPALFLV-GLLSFWAHKHFQPTLPLPPGPPSEFLLGHTRVIPKENAAKV 59
CYP621A1_Fusarium_venenatum                         MIEHYSFAHMLGPALFLT-GLLIFWAHKHFQPTLPLPPGPPSEFLLGHTRVIPKENAAKV 59
CYP621A2_Nectria_haematococca                       -MEFTTAKALIELAIVLA-VMWFALVHRRTQPTLPLPPGPPAEFLLGHTRLIPKENTAAT 58
CYP621A1_Fusarium_vanettenii_77-13-4                -MEFTTAKALIELAIVLA-VMWFALVHRRTQPTLPLPPGPPAEFLLGHTRLIPKENTAAT 58
CYP621A1_Fusarium_solani                            -MEFTTAKALIELAIVLA-VMWFALVHRRTQPTLPLPPGPPAEFLLGHTRLIPKENTAAT 58
CYP621A1_Fusarium_acuminatum_CS5907                 -MDCHVYEALSGLAFILA-VWLLTWAHRGSRHALPLPPGPPSEFLLGHARVIPKENASAV 58
CYP621A1_Fusarium_redolens                          MIEHYPSQALFGLTLAIATILLLAWAHWRSRPTLPLPPGPPSEFLLGHSRVIPRENAAAV 60
CYP621A1_Fusarium_mangiferae                        MIENYPSQALSGLTLAIATILLLAWAHWRSQHTLPLPPGPPSEFLLGHSRVIPKENAAAV 60
CYP621A1_Fusarium_proliferatum_ET1                  MIEHYPSQALSGLTLAIATILLLAWAHWRSQHTLPLPPGPPSEFLLGHFRVIPKENAAAV 60
CYP621A1_Fusarium_fujikuroi_IMI_58289               MIEHYPSQALSGLTLAIATILLLAWAHWRSQHTLPLPPGPPSEFLLGHFRVIPKDNAAAV 60
CYP621A3_Gibberella_moniliformis                    MIEHYLSQALIGLTLAIATVLLFALAHWRSRPTLPLPPGPPSEFLLGHSRVIPKENAAAV 60
CYP621A1_Fusarium_verticillioides_7600              MIEHYLSQALIGLTLAIATVLLFALAHWRSRPTLPLPPGPPSEFLLGHSRVIPKENAAAV 60
CYP621A1_Fusarium_tjaetaba                          MIEHYQSQAFTGLTLAVATGLLLAWAHWRSRPTLPLPPGPPSEFLLGHSRVIPKENAAAV 60
CYP621A1_Fusarium_circinatum_strain_NRRL_25331      MIEHYPSQALSGLTLAIATILLLAWAHWRSRPTLPLPPGPPSEFLLGHSRVIPKENAAAV 60
CYP621A3_Fusarium_oxysporum                         MIEHYPSQALFGLTLAIATILLLAWARWRSRPTLPLPPGPPSEFLLGHSRVIPKENAAAV 60
CYP621A1_Fusarium_odoratissimum_NRRL_54006          MIEHYPSQALSGLMLAIATILLLAWARWRSRPTLPLPPGPPSEFLLGHSRVIPKENAAAV 60
                                                             :    :                    ***.**.:*:*** * ** .::

CYP621B1_Aspergillus_clavatus                       YAKWSRTYNSDILSLRMLTRCVIVVNSVDAAHALLKKPT--AADRPRFALYEIMGWGITL 109
CYP621A1_Fusarium_flagelliforme                     YSRWSREYNSDIIHVRSLGRSTIVLHSAEVAKDILEKKGANFCDRPRFTLLEVMGWGKTL 118
CYP621A1_Fusarium_equesiti                          YSRWSQEYNSDIIHVRSLGRSTMVLHSAKVAKDILEKKGANFCDRPRFTLLEVMGWGKTL 118
CYP621A1_Fusarium_graminearum                       YSRWAKEYNSDIIHVRSLGQSTIVLHSAEVAKDILEKKGANFCDRPRFTLLEVMGWGKTL 119
CYP621A1_Fusarium_pseudograminearum_CS3096          YSRWAKEYNSDIIHVRSLGQSTIVLHSAEVAKDILEKKGANFCDRPRFTLLEVMGWGKTL 119
CYP621A1_Fusarium_poae                              YSRWSKEYNSDIIHVRSLGRSTIVLHSAEVAKDILEKKGANFCDRPRFTLLEVMGWGKTL 119
CYP621A1_Fusarium_venenatum                         YSRWSKEYNSDIIHVRSLGRSTIVLHSAEVAKDILEKKGANFCDRPRFTLLEVMGWGKTL 119
CYP621A2_Nectria_haematococca                       YARWSREYDSDIIHVKSLGRSIVVLNSVEAARDVLEKKGANFCDRPRFTLLEVMGWGKTL 118
CYP621A1_Fusarium_vanettenii_77-13-4                YARWSREYDSDIIHVKSLGRSIVVLNSVEAARDVLEKKGANFCDRPRFTLLEVMGWGKTL 118
CYP621A1_Fusarium_solani                            YARWSREYDSDIIHVKSLGRSIVVLNSVEAARDVLEKKGANFCDRPRFTLLEVMGWGKTL 118
CYP621A1_Fusarium_acuminatum_CS5907                 YAKWSREYDSDIIHFRSLGRSTVVLNSAEAARDILDKKGANFSDRPRFTLLEIMGWGKTV 118
CYP621A1_Fusarium_redolens                          YAKWSKEYNSDIIHVRSLGRSTVVLNIVEVARDILEKKGANFCDRPRFTLLEVMGWGKTL 120
CYP621A1_Fusarium_mangiferae                        YAKWSKEYNSDIIHVRSLGRSTVVLNSADVARDILDKKGANFCDRPRFTLLEVMGWGKTL 120
CYP621A1_Fusarium_proliferatum_ET1                  YAKWSREYNSDIIHVRSLGRSTVVLNSADVARDILDKKGANFCDRPQFTLLEVMGWGKTL 120
CYP621A1_Fusarium_fujikuroi_IMI_58289               YAKWSREYNSDIIHVRSLGRSTVVLNSADVARDILDKKGANFCDRPQFTLLEVMGWGKTL 120
CYP621A3_Gibberella_moniliformis                    YAKWSKEYNSDIIHVRSLGRSTVVLNSADVARDILDKKGANFCDRPRFTLLEVMGWGKTL 120
CYP621A1_Fusarium_verticillioides_7600              YAKWSKEYNSDIIHVRSLGRSTVVLNSADVARDILDKKGANFCDRPRFTLLEVMGWGKTL 120
CYP621A1_Fusarium_tjaetaba                          YAKWSKEYNSDIIHVRSLGRSTVVLNSADVARDILDKKGANFCDRPRFTLLEVMGWGKTL 120
CYP621A1_Fusarium_circinatum_strain_NRRL_25331      YAKWSKDYDSDIIHVRSLGRSTVVLNSADVARDILDKKGANFCDRPRFTLLEVMGWGKTL 120
CYP621A3_Fusarium_oxysporum                         YAKWSKEYNSDIIHVRSLSRSTVVLNSADVARDILDKKGANFCDRPRFTLLKIMGWGKTL 120
CYP621A1_Fusarium_odoratissimum_NRRL_54006          YAKWSKEYNSDIIHVRSLGRSTVVLNSADVARDILDKKGANFCDRPRFTLLEVMGWGKTL 120
                                                    *::*:: *:***: .: * :. :*:: ...*: :*.*     .***:*:* ::**** *:
```

```
CYP621B1_Aspergillus_clavatus                       TFLRSSSPRFKLHRRLLQRSFSPSMCKDYRPIQMQEIRAAVAQIRSHPETWEVSLRRFAI 169
CYP621A1_Fusarium_flagelliforme                     TFLPF-GKCWQMHRKFLQTSFSNTNVRQWHELQRNEAHHTVKNIIETPDTWEKSLRRFAV 177
CYP621A1_Fusarium_equesiti                          TFLPF-GKCWQMHRKFLQTSFSNTNVRQWHELQRNEAHHTVKNIIDTPDTWEKSLRRFAV 177
CYP621A1_Fusarium_graminearum                       TFLPF-GKSWQMHRKFLQTSFSNTNVRQWYNLQMTETHRTVKGIMARPDTWETSLRRFAV 178
CYP621A1_Fusarium_pseudograminearum_CS3096          TFLPF-GKSWQMHRKLLQTSFSNTNVRQWHNLQRTETHRTVKDIMARPGTWETSLRRFAV 178
CYP621A1_Fusarium_poae                              TFMPF-GKSWQMHRKLLQTYFRNTNIRQHHDLQRNEAHRTVKAIMARPGTWQTSLRRFAV 178
CYP621A1_Fusarium_venenatum                         TFLPF-GKSWQMHRKLLQTTFSNTNSRQFYNLQRDEAHRTVKAIMTRPGTWETSLRRFAV 178
CYP621A2_Nectria_haematococca                       TFLPY-GRRWQMHRRLLQTSFSNTNVRQWHKLQITEARRTVRNMMGKPSSWETSLRRLAV 177
CYP621A1_Fusarium_vanettenii_77-13-4                TFLPY-GRRWQMHRRLLQTSFSNTNVRQWHKLQITEARRTVRNMMGKPSSWETSLRRLAV 177
CYP621A1_Fusarium_solani                            TFLPY-GRRWQMHRRLLQTSFSNTNVRQWHKLQITEARRTVRNMMGKPSSWETSLRRLAV 177
CYP621A1_Fusarium_acuminatum_CS5907                 TFLPF-GKRWQMHRKRLQTSFSNTNVRQWHGLQTAEARKTVENMIKKPDTWETSLRRFAV 177
CYP621A1_Fusarium_redolens                          TFLPF-GKRWQMHRKLLQTSFSNTNVRQWHSLQTKEARRTIRNILKKPETWETSLRRLAV 179
CYP621A1_Fusarium_mangiferae                        TFLPF-GERWQMHRKLLQTSFSNTNVRQWHSLQITEARRTIRNILKKPETWETSLRRLAV 179
CYP621A1_Fusarium_proliferatum_ET1                  TFLPF-GERWQMHRKLLQTSFSNTNVRQWHSLQITEARRTIRNIIKKPETWETSLRRLAV 179
CYP621A1_Fusarium_fujikuroi_IMI_58289               TFLPF-GERWQMHRKLLQTSFSNTNVRQWHSLQITEARRTIRNILKKPETWETSLRRLAV 179
CYP621A3_Gibberella_moniliformis                    TFLPF-GQRWQMHRKFLQTSFSNTNVRRWHTLQITEARRTIQNILKKPETWETSLRRLAV 179
CYP621A1_Fusarium_verticillioides_7600              TFLPF-GQRWQMHRKFLQTSFSNTNVRRWHTLQITEARRTIQNILKKPETWETSLRRLAV 179
CYP621A1_Fusarium_tjaetaba                          TFLPF-GESWQMHRKFLQTSFSNTNVRQWHTLQITEARRTIQNILKRPETWETSLRRLAV 179
CYP621A1_Fusarium_circinatum_strain_NRRL_25331      TFLPF-GQRWQMHRKLLQTSFSNTNVRQWHTLQITEARRTVQNMLKKPETWETSLRRLAV 179
CYP621A3_Fusarium_oxysporum                         TFLPF-GERWQMHRKLLQTSFSNTNVRQWHSLQITEARRTIRDILKKPKTWETSLRRLAV 179
CYP621A1_Fusarium_odoratissimum_NRRL_54006          TFLPF-GERWQMHRKLLQTSFSNTNVRQWHSLQITEARRTIRNILKKPKTWETSLRRLAV 179
                                                    **:   .  :::**: **  *  :  :    :*  * : ::  :   * :*: ****:*:

CYP621B1_Aspergillus_clavatus                       AVVMRIGFGMDSAQ-AGGFVDLATAVEEATGRGGVPGFSVVDAVPALRWIPVCVAKRVGM 228
CYP621A1_Fusarium_flagelliforme                     AIVLQVSYGTKVTSDDDPYIRIANDAMYATGNGGVPANSVVDLVPFVRYLPDWLIC---- 233
CYP621A1_Fusarium_equesiti                          AIVLQVSYGTKVTSDDDPYIRIANDAMYATGNGGVPANSVVDLVPFVRYLPDWLIR---- 233
CYP621A1_Fusarium_graminearum                       AIVLQVSYGMEVPSDDDPYIRIANDAMYATGNGGVPANSLVDLVPFVRFLPDWLIN---- 234
CYP621A1_Fusarium_pseudograminearum_CS3096          AIVLQVSYGMEVPSDDDPYIRIANDAMYATGNGGVPANSLVDLVPFVRYLPDWLIN---- 234
CYP621A1_Fusarium_poae                              AIVLQVSYGTEVPSDDDPYIRIANDAMHATGNGGVPANSVVDLVPFVRYLPDWLVN---- 234
CYP621A1_Fusarium_venenatum                         AIVLQVSYGTEVSSDDDPYIRIANDAMYATGNGGVPANSVVDLVPFIRYLPDWLVN---- 234
CYP621A2_Nectria_haematococca                       AIVLQVSYGTEVPKDDDPYIQIANNAMYATGNGGAPANSIVDLIPLARHLPDWIVR---- 233
CYP621A1_Fusarium_vanettenii_77-13-4                AIVLQVSYGTEVPKDDDPYIQIANNAMYATGNGGAPANSIVDLIPLARHLPDWIVR---- 233
CYP621A1_Fusarium_solani                            AIVLQVSYGTEVPKDDDPYIQIANNAMYATGNGGAPANSIVDLIPLARHLPDWIVR---- 233
CYP621A1_Fusarium_acuminatum_CS5907                 AVVLQVSYGTEVLEDDDPCIQIVNDAMYATGNGGVPANSIVDLVPLVRYLPDWIVR---- 233
CYP621A1_Fusarium_redolens                          AIVLQVSYGTQVLKDDDPYIQIANDAMYATGNGGVPANSIVDLVPFVRYLPDCIVR---- 235
CYP621A1_Fusarium_mangiferae                        AIVLQVSYGTQVLEDDDPYIQIANDAMHATGNGGVPANSIVDLVPFVRYLPDCIVR---- 235
CYP621A1_Fusarium_proliferatum_ET1                  AIVLQVSYGTQVLEDDDPYIQIANDAMYATGNGGVPANSIVDLVPFVRYLPDCIVR---- 235
CYP621A1_Fusarium_fujikuroi_IMI_58289               AIVLQVSYGTQVLEDDDPYIQIANDAMYATGNGGVPANSIVDLVPFVRYLPDCIVR---- 235
CYP621A3_Gibberella_moniliformis                    AIVLQVSYGTQVLEDDDPYIQIANDAMYATGNGGVPANSIVDLVPFVRYLPDCIVR---- 235
CYP621A1_Fusarium_verticillioides_7600              AIVLQVSYGTQVLEDDDPYIQIANDAMYATGNGGVPANSIVDLVPFVRYLPDCIVR---- 235
CYP621A1_Fusarium_tjaetaba                          AIVLQVSYGTQVLEDDDPYIQIANDAMYATGNGGVPANSIVDLVPFVRYLPDCIVR---- 235
CYP621A1_Fusarium_circinatum_strain_NRRL_25331      AIVLQVSYGTQVLEDDDPYIQIANDAMYATGNGGVPANSIVDLVPFVRYLPDCIVR---- 235
CYP621A3_Fusarium_oxysporum                         AIVLQVNYGTQVLEDDDPYIQIANDAMYATGNGGVPANSIVDLVPFVRYLPDCIVR---- 235
CYP621A1_Fusarium_odoratissimum_NRRL_54006          AIVLQVSYGTQVLEDDDPYIQIANDAMYATSNGGVPANSIVDLVPFARYLPDCIVR---- 235
                                                    *:*:::.:* .  .  .  : :.. .  **..**.*. *:** :*  * :*  :
```

```
CYP621B1_Aspergillus_clavatus                       LWGLVHAQQMRGAVEEFHNGPWGMMEEKL------RAGTGETGSSFL----------GMH 272
CYP621A1_Fusarium_flagelliforme                     DWSLRFARQWQWAITKLHDVPFAAAQTEHLTYLLSRNGHSSTNTSLAHQLLQLYDYNELQ 293
CYP621A1_Fusarium_equesiti                          DWSLRFARQWQWAITKLHDVPFAAAQAEHLTYLLSRNGHSSTNTSLAHQLLQLYDCNESQ 293
CYP621A1_Fusarium_graminearum                       DWSLRFARQWRWAITKLHDVPFAAAQAER-----LCNNKYPANTSLAHQLLQSYEHNELQ 289
CYP621A1_Fusarium_pseudograminearum_CS3096          DWSLRFARQWRWAITKLHDVPFAAAQAER-----LCTDKSSANTSLAYQLLQSYEHNELQ 289
CYP621A1_Fusarium_poae                              DWSLQFARQWRWAITKLHDVPFTAAQAKR-----SCNDDYPANTSLAHQLLRSYEYNKLQ 289
CYP621A1_Fusarium_venenatum                         DWSLRFARQWRWAITKLHEVPFAAAQAER-----LCNDEFSANTSLAHQLLRSYEYNQSQ 288
CYP621A2_Nectria_haematococca                       DWSLRFARQWRWAIQKLHDVPFAAAQSEH--------DGCSDNKSLAHELLRQYRRNEEN 285
CYP621A1_Fusarium_vanettenii_77-13-4                DWSLRFARQWRWAIQKLHDVPFAAAQSEH--------DGCSDNKSLAHELLRQYRRNEEN 285
CYP621A1_Fusarium_solani                            DWSLRFARQWRWAIQKLHDVPFAAAQSEH--------DGCSDNKSLAHELLRQYRRNEEN 285
CYP621A1_Fusarium_acuminatum_CS5907                 DWSLRFARNWRWAIQKLHDVPFTAAQAEH-----LCNGHHSENKSLAHQLLREYEDKEAH 288
CYP621A1_Fusarium_redolens                          DLSLRFARQWRWAIKKIHNVPFAAAQAEY------HRGDGHGNTSLAHQLLREYKEKEFH 289
CYP621A1_Fusarium_mangiferae                        DWSLRFARQWRWAIKRLHDVPFAAAQAEY------HRYDHHRNTSLAHHLLREYKDKESH 289
CYP621A1_Fusarium_proliferatum_ET1                  DWSLRFARQWRWAIKKLHDIPFAAAQAEY------HRYDHHRNTSLAHNLLREYKDKESR 289
CYP621A1_Fusarium_fujikuroi_IMI_58289               DWSLRFARQWRWAIRKLHDIPFAAAQAEY------HRYDHHRNTSLAHNLLREYKDKESR 289
CYP621A3_Gibberella_moniliformis                    DWSLRFARQWRWAIKKLHDVPFAAAQAEN------HRYDYYTNTSLAHHLLREYKDKESR 289
CYP621A1_Fusarium_verticillioides_7600              DWSLRFARQWRWAIKKLHDVPFAAAQAEN------HRYDYYTNTSLAHHLLREYKDKESR 289
CYP621A1_Fusarium_tjaetaba                          DWSLRFARQWRWAIKKLHDVPFAAAQAEN------HRYDHYTDTSLAHHLLREYKDKESR 289
CYP621A1_Fusarium_circinatum_strain_NRRL_25331      DWSLRFARQWRWAIKKLHDVPFAAAQAEY------HRYDRHRNKSLAHHLLREYKENESR 289
CYP621A3_Fusarium_oxysporum                         DWSLRFARQWRWAIKKLHDVPLAAAQAEY------HRDDHYRNTSLTHLLLREYKDKEFR 289
CYP621A1_Fusarium_odoratissimum_NRRL_54006          DWSLRFARQWRWAIKKLHDVPFAAAQAEY------HRDDHHRNTSLAHHLLREYKDKEFR 289
                                                      .* .*:: : *: .:*: *    :                ..*:             .

CYP621B1_Aspergillus_clavatus                       LREGKVGVEDLKGAAGTIAIAGGNTTFATIVVCILNLMLQPGVQARARAELDGVLGVDAR 332
CYP621A1_Fusarium_flagelliforme                     GQKQEWALDDIKGAAGAVFIAGTDTTWATCVIFILNMVLHPEIQKKAQAELDTVIGPG-- 351
CYP621A1_Fusarium_equesiti                          GQKQEWALDDIKGAAGAVFIAGTDTTWATCVIFILNMVLHPEIQKKAQAELDAVIGSG-- 351
CYP621A1_Fusarium_graminearum                       GNKQDWSLDDIKGAAGAVFIAGADTTWATCVIFVLNMVLHPEIQKKAQDELDAVLRPG-- 347
CYP621A1_Fusarium_pseudograminearum_CS3096          GNKQDWSLDDIKGAAGAVFIAGADTTWATCVIFVLNMVLHPEIQKKAQDELDAVLRPG-- 347
CYP621A1_Fusarium_poae                              GKNQEWSLDDIKGAAGAVFIAGADTTWATCVIFVLNMVLHPEIQKKAQDELDAVIGPG-- 347
CYP621A1_Fusarium_venenatum                         GKNQKWSLDDIKGAAGAVFIAGADTTWATCVIFVLNMVLHPEIQKKAQDELDAVIGPG-- 346
CYP621A2_Nectria_haematococca                       GQEQEWSLDDIKGAAGAVFIAGADTTWATCVIFILNMVLHPEIQEKARSQLDSVIGPD-- 343
CYP621A1_Fusarium_vanettenii_77-13-4                GQEQEWSLDDIKGAAGAVFIAGADTTWATCVIFILNMVLHPEIQEKARSQLDSVIGPD-- 343
CYP621A1_Fusarium_solani                            GQEQEWSLDDIKGAAGAVFIAGADTTWATCVIFILNMVLHPEIQEKARSQLDSVIGPD-- 343
CYP621A1_Fusarium_acuminatum_CS5907                 GQQKGWSLDDIKGAAGAVLIAGADTTWATCSIFILNMVLHPMIQKKAQKELDGVVGLD-- 346
CYP621A1_Fusarium_redolens                          GQEQQWSLDDIKGAAGAVFIAGADTTWATCVIFILNMVLHPEIQEKAHKELDTVIGSD-- 347
CYP621A1_Fusarium_mangiferae                        GQEQQWSLDDIKGAAGAVFIAGADTTWATCVIFILNMVLHPEIQDKAQQELDTVIGFD-- 347
CYP621A1_Fusarium_proliferatum_ET1                  GQEQQWSLDDIKGAAGAVFIAGADTTWATCVIFILNMVLHPEIQDKAQQELDTLIGFD-- 347
CYP621A1_Fusarium_fujikuroi_IMI_58289               GQEQQWSLDDIKGAAGAVFIAGADTTWATCVIFILNMVLHPEIQDKAQQELDTVIGFD-- 347
CYP621A3_Gibberella_moniliformis                    GQEQQWSLDDIKGAAGAVFIAGTDTTWATCVIFILNMVLHPEIQEKAQQELDAVIGSD-- 347
CYP621A1_Fusarium_verticillioides_7600              GQEQQWSLDDIKGAAGAVFIAGTDTTWATCVIFILNMVLHPEIQEKAQQELDAVIGSD-- 347
CYP621A1_Fusarium_tjaetaba                          GQEQQWSLDDIKGAAGAVFIAGADTTWATCVIFILNMVLHPEIQEKAQQELDAVVGSD-- 347
CYP621A1_Fusarium_circinatum_strain_NRRL_25331      GQEQQWSLDDIKGAAGAVFIAGADTTWATCVIFILNMVLHPEIQEKAQQELDAVIGSN-- 347
CYP621A3_Fusarium_oxysporum                         GQEQQWSLDDIKGAAGAVFIAGADTTWATCVIFVLNMVLHPEIQEKAQQELDAVIGTD-- 347
CYP621A1_Fusarium_odoratissimum_NRRL_54006          GQEQQWSLDDIKGAAGAVFIAGADTTWATCVIFILNMVLHPEIQEKAQQELDAVIGTD-- 347
                                                     .:   .::*:*****:: *** :**:**  : :**::*:* :* :*: :** ::  .
```

```
CYP621B1_Aspergillus_clavatus                       GLPLRLPTFEDRERLPFLERVIQETTRWAPLSPLGIPHAMSAEESVGGLTIPRGAVVYAN 392
CYP621A1_Fusarium_flagelliforme                     ----KLPRFSNRPRLPYIEHIVQETYRWSPLAPLGIPHKSLHDDMYQGMFIPKGTVVYAN 407
CYP621A1_Fusarium_equesiti                          ----KLPRFSDRPRLPYIEHIVQETYRWSPLAPLGIPHKSLHDDVYQGMFIPKGTVVYAN 407
CYP621A1_Fusarium_graminearum                       ----KLPEFSDRPSLPYVEHIVQEIYRWSPLAPLGIPHKSLHDDVYHGMFIPKGTVVYAN 403
CYP621A1_Fusarium_pseudograminearum_CS3096          ----KLPEFSDRPSLPYVEHIVQEIYRWSPLAPLGIPHKSLHDDVYHGMFIPKGTVVYAN 403
CYP621A1_Fusarium_poae                              ----KLPEFSDRPSLPYIEHIVQEIYRWSPLAPLGIPHKSLHDDVYQGMLIPKGTVVYAN 403
CYP621A1_Fusarium_venenatum                         ----KLPEFSDRSYLPYIEHIVQEIYRWSPLAPLGIPHKSLHDDVYQGMFIPKGTVVYAN 402
CYP621A2_Nectria_haematococca                       ----RLPNFSDRASLPYIEHIVQEIYRWSPLAPLGIPHKSLQDDVYQGMFIPKGTVVYAN 399
CYP621A1_Fusarium_vanettenii_77-13-4                ----RLPNFSDRASLPYIEHIVQEIYRWSPLAPLGIPHKSLQDDVYQGMFIPKGTVVYAN 399
CYP621A1_Fusarium_solani                            ----RLPNFSDRASLPYIEHIVQEIYRWSPLAPLGIPHKSLQDDVYQGMFIPKGTVVYAN 399
CYP621A1_Fusarium_acuminatum_CS5907                 ----RLPELSDRPFLPHIEHIVQEIYRWSPLAPLGIPHKSLQDDVYQGMFIPKGTVVYAN 402
CYP621A1_Fusarium_redolens                          ----RLPGFSDRPALVYIEHIVHGIYRWSPLAPLGIPHKSLHDDIYKGMLIPKGTVVYAN 403
CYP621A1_Fusarium_mangiferae                        ----RLPDFSDRPALVYIEHIVQEIYRWSPLAPLGIPHKSLHDDIYKGMLIPKGTVVYAN 403
CYP621A1_Fusarium_proliferatum_ET1                  ----KLPDFSDRPALVYIEHVVQEIYRWSPLAPLGIPHKSLHDDIYKGMHIPKGTVVYAN 403
CYP621A1_Fusarium_fujikuroi_IMI_58289               ----KLPDFSDRPALVYIEHVVQEIYRWSPLAPLGIPHKSLHDDIYKGMHIPKGTVVYAN 403
CYP621A3_Gibberella_moniliformis                    ----RLPDFSDRPALVYIEHIVQEIYRWSPLAPLGIPHKSLHDDIYKGMLIPKGTVVYAN 403
CYP621A1_Fusarium_verticillioides_7600              ----RLPDFSDRPALVYIEHIVQEIYRWSPLAPLGIPHKSLHDDIYKGMLIPKGTVVYAN 403
CYP621A1_Fusarium_tjaetaba                          ----RLPDFSDRPALAYIEHIVQEIYRWSPLAPLGIPHKSLHDDIYKGMLIPKGTVVYAN 403
CYP621A1_Fusarium_circinatum_strain_NRRL_25331      ----RLPDFSDRPALVYIEHIVQEIYRWSPLAPLGIPHKSLHDDIYKGMLIPKGTVVYAN 403
CYP621A3_Fusarium_oxysporum                         ----RLPDFSDRPALVYIEHIVQEIYRWSPLAPLGIPHKSLHDDIYKGMLIPKGTVVYAN 403
CYP621A1_Fusarium_odoratissimum_NRRL_54006          ----RLPDFSDRPALVYIEHIVQEIYRWSPLAPLGIPHKSLHDDIYKGMLIPKGTVVYAN 403
                                                        :** :.:*  * .:*::::   **:**:******    ::   *: **:*:*****

CYP621B1_Aspergillus_clavatus                       AWAMTHDERVYAEPERFDPDRYL------RGEPLPEGPFGFGRRVCPGQHLALTGVYIAM 446
CYP621A1_Fusarium_flagelliforme                     TYAIAHDERVYENPQDFNPDRYG------GGEPYPVGNFGFGRRVCVGRFLADNSVWIMV 461
CYP621A1_Fusarium_equesiti                          AYAIAHDERVYQNPHDFNPDRYG------GGEPYPVGNFGFGRRVCVGRFLADNSVWIMV 461
CYP621A1_Fusarium_graminearum                       SYAIAHDERVYKSPHEFNPDRYG------AGEPYPVGNFGFGRRICVGRFLAGNSVWIMV 457
CYP621A1_Fusarium_pseudograminearum_CS3096          SYAIAHDERVYKSPHEFNPDRYG------AGEPYPVGNFGFGRRICVGRFLAGNSVWIMV 457
CYP621A1_Fusarium_poae                              SYAIAHDERVYKAPHDFNPDRYS------AGEPYPVGNFGFGRRICVGRFLADNSVWIMV 457
CYP621A1_Fusarium_venenatum                         SYAIAHDERVYKAPHDFNPDRYS------AGEPYPVGNFGFGRRICVGRFLADNSVWVMV 456
CYP621A2_Nectria_haematococca                       AHAMAHDERIYRAPHDFNPDRYEPLVNGGAGEPFPVGNFGFGRRVCVGRFLADNSVWIMV 459
CYP621A1_Fusarium_vanettenii_77-13-4                AHAMAHDERIYRAPHDFNPDRYEPLVNGGAGEPFPVGNFGFGRRVCVGRFLADNSVWIMV 459
CYP621A1_Fusarium_solani                            AHAMAHDERIYRAPHDFNPDRYEPLVNGGAGEPFPVGNFGFGRRVCVGRFLADNSVWIMV 459
CYP621A1_Fusarium_acuminatum_CS5907                 AHAIAHDERVYRDPHKFNPDRYE------AGEPFPVGNFGFGRRICVGRFLADNSVWIMV 456
CYP621A1_Fusarium_redolens                          AYTMAHDGRVYKNPHDFNPNRYE------ARELFPVGNFGFGRRVCVGRFLADNSVWIMV 457
CYP621A1_Fusarium_mangiferae                        AYAMAHDERVYKDPHDFNPDRYE------AGEPFPVGNFGFGRRVCVGRFLADNSVWIMV 457
CYP621A1_Fusarium_proliferatum_ET1                  AYAMAHDERVYKDPHDFNPDRYE------AGEPFPVGNFGFGRRVCVGRFLADNSVWIMV 457
CYP621A1_Fusarium_fujikuroi_IMI_58289               AYAMAHDERVYKDPHDFNPDRYE------AGEPFPVGNFGFGRRVCVGRFLADNSVWIMV 457
CYP621A3_Gibberella_moniliformis                    AYAIAHDERVYKNPHNFNPDRYE------AGEPFPVGNFGFGRRVCVGRFLADNSVWIMV 457
CYP621A1_Fusarium_verticillioides_7600              AYAIAHDERVYKNPHNFNPDRYE------AGEPFPVGNFGFGRRVCVGRFLADNSVWIMV 457
CYP621A1_Fusarium_tjaetaba                          AYAMAHDERVYKDPHDFNPDRYE------AGEPFPVGNFGFGRRVCVGRFLADNSVWIMV 457
CYP621A1_Fusarium_circinatum_strain_NRRL_25331      AYAMAHDERVYKNPHDFNPDRYE------AGEPFPVGNFGFGRRVCVGRFLADNSVWIMV 457
CYP621A3_Fusarium_oxysporum                         AYAMAHDERVYKNPHDFNPDRYE------AGEPFPVGNFGFGRRVCVGRFLADNSVWIMV 457
CYP621A1_Fusarium_odoratissimum_NRRL_54006          AYAMAHDERVYKNPHDFNPDRYE------AGEPFPVGNFGFGRRVCVGRFLADNSVWIMV 457
                                                    : :::** *:*  *. *:*:**         *  * * ******:* *:.** ..*:: :
```

```
CYP621B1_Aspergillus_clavatus                       ATLLATVSWRCPVDEAGREKRPEVQFSDGLSGVPDRFECEMQARDAESKEVL*-----   498
CYP621A1_Fusarium_flagelliforme                     ATMLSTLEFCKKVAQDGTPIEPRVQFTNGGTCHPEHFDCVIKPRTSKAEALFNAN---   516
CYP621A1_Fusarium_equesiti                          ATMLSTLEFCKKAAQDGTPIEPRVQFTNGGTCHPEHFDCVIKPRTSKAEALFNAN*--   516
CYP621A1_Fusarium_graminearum                       ATMLSTLQFCKKVAQDGTPIEPRVQFTNGGTCHPEHFDCVIKPRNATATALINAS*--   512
CYP621A1_Fusarium_pseudograminearum_CS3096          ATMLSTLQFCKKVAQDGTLIEPRVQFTNGGTCHPEHFDCVIKPRNSTATAFINAS*--   512
CYP621A1_Fusarium_poae                              ATMLSTLNFCKKVAQDGRPIEPRVRFTNGGTCHPEHFDCDIRRRNSTATVLINAS*--   512
CYP621A1_Fusarium_venenatum                         ATMLSTLNFCKQVAQDGRPIEPRVRFTNGGTCHPEHFDCDIRPRNSAATALINAS*--   511
CYP621A2_Nectria_haematococca                       ATMLATLEFRKKMGPDGSPIEPRVQFTNGGTCHPEHFECDIRPRSHKAAELIGANHD*   516
CYP621A1_Fusarium_vanettenii_77-13-4                ATMLATLEFRKKMGPDGSPIEPRVQFTNGGTCHPEHFECDIRPRSHKAAELIGANHD-   516
CYP621A1_Fusarium_solani                            ATMLATLEFRKKMGPDGSPIEPRVQFTNGGTCHPEHFECDIRPRSHKAAELIGANHD*   516
CYP621A1_Fusarium_acuminatum_CS5907                 ATMLSTLQFGKKVDLDGAPIEPRVQFTNGGTCHPEHFGCVIKPRNSNSATLVGVSSE*   513
CYP621A1_Fusarium_redolens                          ATMLSALQFCK-MSSDGKPIEPRVRFTNGGTCHPEHFDCVIKPRNLKAEALVESD*--   511
CYP621A1_Fusarium_mangiferae                        ATMLSVLRFCKKMSTDGKLIEPRVRFTNGGTCHPEHFDCVIKPRSLAAEALVNSNSE-   514
CYP621A1_Fusarium_proliferatum_ET1                  ATMLSVLRFCKKMSSDGKLIEPRVRFTNGGTCHPEHFDCVIKPRSLAAEALVNSDSE*   514
CYP621A1_Fusarium_fujikuroi_IMI_58289               ATMLSVLRFCKKMSSDGKLIEPRVRFTNGGTCHPEHFDCVIKPRSLAAEALVNSDSE-   514
CYP621A3_Gibberella_moniliformis                    ATMLSVLRFCKKMSSDGKPIEPRVQFTNGGTCHPEHFDCVIRPRSLATEALVNSD*--   512
CYP621A1_Fusarium_verticillioides_7600              ATMLSVLRFCKKMSSDGKPIEPRVQFTNGGTCHPEHFDCVIRPRSLATEALVNSD*--   512
CYP621A1_Fusarium_tjaetaba                          ATMLSVLRFCKKMSSDGKPIEPRVRFTNGGTCHPEHFDCVIKPRSLAAEALVNSD*--   512
CYP621A1_Fusarium_circinatum_strain_NRRL_25331      ATMLSVLRFCKKISPDGKPIEPRVRFTNGGTCHPEHFDCVIKPRSLAAEALVNSD*--   512
CYP621A3_Fusarium_oxysporum                         ATMLSVLRFCKKMSSDGKPIEPRVRFTNGGTCHPEHFDCVIKSRSLEAEALVKSD*--   512
CYP621A1_Fusarium_odoratissimum_NRRL_54006          ATMLSVLRFCKKMSSDGKPIEPRVRFTNGGTCHPEHFDCVIKPRSLEAEALVKSD---   512
                                                    **:*:.: :       *   .*.*:*::* :  *::* * :: *   :  ..
```
