# Supplementary material for: The Ultimate Micro-Exon: A Single Nucleotide Exon Is Required to Assemble Cytochrome P450 CYP621A Orthologs from Fusarium Species
Source: Int J Mol Sci. 2026 Feb 19;27(4):1979. doi: 10.3390/ijms27041979 (PMC12940212; doi:10.3390/ijms27041979)
Supplement: Supplementary file 1 [file ijms-27-01979-s001.zip › Figure S2.pdf]

Figure S2: Manual curation and accurate prediction of CYP621 members from different *Fusarium* species (A) and their multiple sequence alignment highlighting the PKG region (B).

A

```
>CYP621A1 Fusarium graminearum NZ_AACM02000168.1
This sequence is shifted 5 base pairs 5’ from the annotated sequence NW_059885.1 with
the gene model FG05113.1. Note: no good phase 0 boundary can be found near CNNK. The
next available boundary removes the conserved sequence SLAHQ from the protein. If the
genomic sequence is correct, this gene may not be expressed for lack of an intron
boundary, or it may be missing 14 amino acids CNNKYPANTSLAHQ in the middle of the protein.

74806 MIELYSFAHMLGPALVFTSLLIFWAHTHFQPTLPLPPGPPSEFLLGHTRVIPKENAAEVYSRWAKEY (1) 74605
74549 NSDIIHVRSLGQSTIVLHSAEVAKDILEKKGANFCDRPRFTLLEV (2) 74417
74340 MGWGKTLTFLPFGKSWQMHRKFLQTSFSNTNVRQWYNLQMTETHRTVKGIMARPDTWET
      SLRRFAVAIVLQVSYGMEVPSDDDPYIRIANDAMYATGNGGVPANSLVDLVPF (1) 74003
73943 VRFLPDWLINDWSLRFARQWRWAITKLHDVPFAAAQAER (0) 73828
73751 (?) CNNKYPANTSLAHQLLQSYEHNELQGNKQDWSLDDIKGAAGAVFIAGADT (0) 73602
73549 TWATCVIFVLNMVLHPEIQKKAQDELDAVLRPGKLPEFSDRPSLPYVEHIVQEIYR (2) 73383
73317 WSPLAPL (1) 73294
73221 GIPHKSLHDDVYHGMFIPK (1) 73165
73095 G (2) 73095
73025 TVVYANSYAIAHDERVYKSPHEFNPDRYGAGEPYPVGNFGFGRR (2) 72894
72884 ICVGRFLAGNSVWIMVATMLSTLQFCKKVAQDGTPIEPRVQFTNGGTC (2) 72691
72529 HPEHFDCVIKPRNATATALINAS* 72457

>CYP621A2 Nectria haematococca
JGI gene model e_gw1.5.1476.1 Necha1/scaffold_5:782235-784633

782235 MEFTTAKALIELAIVLAVMWFALVHRRTQPTLPLPPGPPAEFLLGHTRLIPKENTAATYARWSREY (1) 782433
782503 DSDIIHVKSLGRSIVVLNSVEAARDVLEKKGANFCDRPRFTLLEV (2) 782635
782713 MGWGKTLTFLPYGRRWQMHRRLLQTSFSNTNVRQWHKLQITEARRTVRNMMGKPSSWET
       SLRRLAVAIVLQVSYGTEVPKDDDPYIQIANNAMYATGNGGAPANSIVDLIPL (1) 783050
783117 ARHLPDWIVRDWSLRFARQWRWAIQKLHDVPFAAAQSEH (0) 783232
783312 DGCSDNKSLAHELLRQYRRNEENGQEQEWSLDDIKGAAGAVFIAGADT (0) 783455
783513 TWATCVIFILNMVLHPEIQEKARSQLDSVIGPDRLPNFSDRASLPYIEHIVQEIYR (2) 783679
783741 WSPLAPL (1) 783763
783841 GIPHKSLQDDVYQGMFIPK (1) 783897
783951 G (2) 783951
784010 TVVYANAHAMAHDERIYRAPHDFNPDRYEPLVNGGAGEPFPVGNFGFGRR (2) 784160
784231 VCVGRFLADNSVWIMVATMLATLEFRKKMGPDGSPIEPRVQFTNGGTC (2) 784374
784555 HPEHFECDIRPRSHKAAELIGANHD* 784633

>CYP621A3 Gibberella moniliformis
AAIM02000074.1 79% to CYP621A2 Nectria haematococca
Also called Fusarium verticillioides gene model FVEG_06100

121996 MIEHYLSQALIGLTLAIATVLLFALAHWRSRPTLPLPPGPPSEFLLGHSRVIPKENAAAVYAKWSKEY (1) 121791
121732 NSDIIHVRSLGRSTVVLNSADVARDILDKKGANFCDRPRFTLLEV (2) 121600
121523 MGWGKTLTFLPFGQRWQMHRKFLQTSFSNTNVRRWHTLQITEARRTIQNILKKPETWETS 5669
       LRRLAVAIVLQVSYGTQVLEDDDPYIQIANDAMYATGNGGVPANSIVDLVPF (1) 121186
121122 VRYLPDCIVRDWSLRFARQWRWAIKKLHDVPFAAAQAEN (0) 121007
120922 HRYDYYTNTSLAHHLLREYKDKESRGQEQQWSLDDIKGAAGAVFIAGTDT (0) 120773
120718 TWATCVIFILNMVLHPEIQEKAQQELDAVIGSDRLPDFSDRPALVYIEHIVQEIYR (2) 120552
120491 WSPLAPL (1) 120469
120410 GIPHKSLHDDIYKGMLIPK (1) 120354
120296 G (2) 120296
120223 TVVYANAYAIAHDERVYKNPHNFNPDRYEAGEPFPVGNFGFGRR (2) 120092
120018 VCVGRFLADNSVWIMVATMLSVLRFCKKMSSDGKPIEPRVQFTNGGTC (2) 119875
119740 HPEHFDCVIRPRSLATEALVNSD* 119668

>CYP621A3  Fusarium oxysporum AAXH01000542 gene model FOXG_08569
93% to CYP621A3 Gibberella moniliformis

41682 MIEHYPSQALFGLTLAIATILLLAWARWRSRPTLPLPPGPPSEFLLGHSRVIPKENAAAVYAKWSKEY (1) 41885
41946 NSDIIHVRSLSRSTVVLNSADVARDILDKKGANFCDRPRFTLLKI (2) 42078
42151 MGWGKTLTFLPFGERWQMHRKLLQTSFSNTNVRQWHSLQITEARRTIRDILKKPKTWETS
      LRRLAVAIVLQVNYGTQVLEDDDPYIQIANDAMYATGNGGVPANSIVDLVPF (1) 42488
42552 VRYLPDCIVRDWSLRFARQWRWAIKKLHDVPLAAAQAEY (0) 42667
42750 HRDDHYRNTSLTHLLLREYKDKEFRGQEQQWSLDDIKGAAGAVFIAGADT (0) 42902
42957 TWATCVIFVLNMVLHPEIQEKAQQELDAVIGTDRLPDFSDRPALVYIEHIVQEIYR (2) 43123
43186 WSPLAPL (1) 43208
43267 GIPHKSLHDDIYKGMLIPK (1) 43323
43381 G (2) 43381
43454 TVVYANAYAMAHDERVYKNPHDFNPDRYEAGEPFPVGNFGFGRR (2) 43585
43660 VCVGRFLADNSVWIMVATMLSVLRFCKKMSSDGKPIEPRVRFTNGGTC (2) 43803
43938  HPEHFDCVIKSRSLEAEALVKSD*  44010

>CYP621B1 Aspergillus clavatus
XM_001274332, locus tag ACLA_013420, from the genomic sequence NW_001517104
complement(1359061..1360557) no introns
43% identical to CYP621A3 Gibberella moniliformis

MESLILLVLIILPASVLIYKRLTPRNLPPSPPGDFFLGHLRRIPSSHAEYQYAKWSRTYNSDILSLRMLTRCVIVVNSVD
AAHALLKKPTAADRPRFALYEIMGWGITLTFLRSSSPRFKLHRRLLQRSFSPSMCKDYRPIQMQEIRAAVAQIRSHPETW
EVSLRRFAIAVVMRIGFGMDSAQAGGFVDLATAVEEATGRGGVPGFSVVDAVPALRWIPVCVAKRVGMLWGLVHAQQMRG
AVEEFHNGPWGMMEEKLRAGTGETGSSFLGMHLREGKVGVEDLKGAAGTIAIAGGNTTFATIVVCILNLMLQPGVQARAR
AELDGVLGVDARGLPLRLPTFEDRERLPFLERVIQETTRWAPLSPLGIPHAMSAEESVGGLTIPRGAVVYANAWAMTHDE
RVYAEPERFDPDRYLRGEPLPEGPFGFGRRVCPGQHLALTGVYIAMATLLATVSWRCPVDEAGREKRPEVQFSDGLSGVP
DRFECEMQARDAESKEVL*
```

B

```
621A3 F. oxysporum      MIEHYPSQALFGLTLAIATILLLAWARWRSRPTLPLPPGPPSEFLLGHSRVIPKENAAAV 60
621A3 G. moniliformis   MIEHYLSQALIGLTLAIATVLLFALAHWRSRPTLPLPPGPPSEFLLGHSRVIPKENAAAV 60
621A1 F. graminearum    -MIELYSFAHMLGPALVFTSLLIFWAHTHFQPTLPLPPGPPSEFLLGHTRVIPKENAAEV 59
621A2 N. haematococca   --MEFTTAKALIELAIVLAVMWFALVHRRTQPTLPLPPGPPAEFLLGHTRLIPKENTAAT 58
621B1 A. clavatus       ---------MESLILLVLIILPASVLIYKRLTPRNLPPSPPGDFFLGHLRRIPSSHAEYQ 51
                                        :   :       :  ..  ***.**.:*:*** * **..::

621A3 F. oxysporum      YAKWSKEYNSDIIHVRSLSRSTVVLNSADVARDILDKKGANFCDRPRFTLLKIMGWGKTL 120
621A3 G. moniliformis   YAKWSKEYNSDIIHVRSLGRSTVVLNSADVARDILDKKGANFCDRPRFTLLEVMGWGKTL 120
621A1 F. graminearum    YSRWAKEYNSDIIHVRSLGQSTIVLHSAEVAKDILEKKGANFCDRPRFTLLEVMGWGKTL 119
621A2 N. haematococca   YARWSREYDSDIIHVKSLGRSIVVLNSVEAARDVLEKKGANFCDRPRFTLLEVMGWGKTL 118
621B1 A. clavatus       YAKWSRTYNSDILSLRMLTRCVIVVNSVDAAHALLKKP--TAADRPRFALYEIMGWGITL 109
                        *::*:: *:***: :: * :. :*::*.:.*: :*.*   . .*****:* ::**** **

621A3 F. oxysporum      TFLPFG-ERWQMHRKLLQTSFSNTNVRQWHSLQITEARRTIRDILKKPKTWETSLRRLAV 179
621A3 G. moniliformis   TFLPFG-QRWQMHRKFLQTSFSNTNVRRWHTLQITEARRTIQNILKKPETWETSLRRLAV 179
621A1 F. graminearum    TFLPFG-KSWQMHRKFLQTSFSNTNVRQWYNLQMTETHRTVKGIMARPDTWETSLRRFAV 178
621A2 N. haematococca   TFLPYG-RRWQMHRRLLQTSFSNTNVRQWHKLQITEARRTVRNMMGKPSSWETSLRRLAV 177
621B1 A. clavatus       TFLRSSSPRFKLHRRLLQRSFSPSMCKDYRPIQMQEIRAAVAQIRSHPETWEVSLRRFAI 169
                        ***  .   :::**::** *** :  : :  :*: * : ::  :  :*.:**.****:*:

621A3 F. oxysporum      AIVLQVNYGTQVLEDDDPYIQIANDAMYATGNGGVPANSIVDLVPFVRYLPDCIVR---- 235
621A3 G. moniliformis   AIVLQVSYGTQVLEDDDPYIQIANDAMYATGNGGVPANSIVDLVPFVRYLPDCIVR---- 235
621A1 F. graminearum    AIVLQVSYGMEVPSDDDPYIRIANDAMYATGNGGVPANSLVDLVPFVRFLPDWLIN---- 234
621A2 N. haematococca   AIVLQVSYGTEVPKDDDPYIQIANNAMYATGNGGAPANSIVDLIPLARHLPDWIVR---- 233
621B1 A. clavatus       AVVMRIGFGMDSAQAGG-FVDLATAVEEATGRGGVPGFSVVDAVPALRWIPVCVAKRVGM 228
                        *:*:::.:* :  . .. :: :*. .  ***.**.*. *:** :*  * :*  : .

621A3 F. oxysporum      DWSLRFARQWRWAIKKLHDVPLAAAQAEYHRDDHYRNTSLTHLLLREYKDKEFRGQEQQW 295
621A3 G. moniliformis   DWSLRFARQWRWAIKKLHDVPFAAAQAENHRYDYYTNTSLAHHLLREYKDKESRGQEQQW 295
621A1 F. graminearum    DWSLRFARQWRWAITKLHDVPFAAAQAERCNNKYPANTSLAHQLLQSYEHNELQGNKQDW 294
621A2 N. haematococca   DWSLRFARQWRWAIQKLHDVPFAAAQSEHDG--CSDNKSLAHELLRQYRRNEENGQEQEW 291
621B1 A. clavatus       LWGLVHAQQMRGAVEEFHNGPWGMMEEKLRAGTGETGSSFLGMHLREGKVG--------- 279
                         *.* .*:* * *: ::*: * .  : :        ..*:    *:. .

621A3 F. oxysporum      SLDDIKGAAGAVFIAGADTTWATCVIFVLNMVLHPEIQEKAQQELDAVIGTDR------L 349
621A3 G. moniliformis   SLDDIKGAAGAVFIAGTDTTWATCVIFILNMVLHPEIQEKAQQELDAVIGSDR------L 349
621A1 F. graminearum    SLDDIKGAAGAVFIAGADTTWATCVIFVLNMVLHPEIQKKAQDELDAVLRPGK------L 348
621A2 N. haematococca   SLDDIKGAAGAVFIAGADTTWATCVIFILNMVLHPEIQEKARSQLDSVIGPDR------L 345
621B1 A. clavatus       -VEDLKGAAGTIAIAGGNTTFATIVVCILNLMLQPGVQARARAELDGVLGVDARGLPLRL 338
                         ::*:*****:: *** :**:** *: :**::*:* :* :*: :**.*:  .       *

621A3 F. oxysporum      PDFSDRPALVYIEHIVQEIYRWSPLAPLGIPHKSLHDDIYKGMLIPKGTVVYANAYAMAH 409
621A3 G. moniliformis   PDFSDRPALVYIEHIVQEIYRWSPLAPLGIPHKSLHDDIYKGMLIPKGTVVYANAYAIAH 409
621A1 F. graminearum    PEFSDRPSLPYVEHIVQEIYRWSPLAPLGIPHKSLHDDVYHGMFIPKGTVVYANSYAIAH 408
621A2 N. haematococca   PNFSDRASLPYIEHIVQEIYRWSPLAPLGIPHKSLQDDVYQGMFIPKGTVVYANAHAMAH 405
621B1 A. clavatus       PTFEDRERLPFLERVIQETTRWAPLSPLGIPHAMSAEESVGGLTIPRGAVVYANAWAMTH 398
                        * *.**  * ::*:::**  **:**:******    ::   *: **:*:*****: *::*

621A3 F. oxysporum      DERVYKNPHDFNPDRYE------AGEPFPVGNFGFGRRVCVGRFLADNSVWIMVATMLSV 463
621A3 G. moniliformis   DERVYKNPHNFNPDRYE------AGEPFPVGNFGFGRRVCVGRFLADNSVWIMVATMLSV 463
621A1 F. graminearum    DERVYKSPHEFNPDRYG------AGEPYPVGNFGFGRRICVGRFLAGNSVWIMVATMLST 462
621A2 N. haematococca   DERIYRAPHDFNPDRYEPLVNGGAGEPFPVGNFGFGRRVCVGRFLADNSVWIMVATMLAT 465
621B1 A. clavatus       DERVYAEPERFDPDRYLR------GEPLPEGPFGFGRRVCPGQHLALTGVYIAMATLLAT 452
                        ***:*  *. *:****        *** * * ******:* *:.** ..*:* :**:*:.

621A3 F. oxysporum      LRFCKKMSSDGKPIEPRVRFTNGGTWYIISTVPMACFALTHSLQ------- 507
621A3 G. moniliformis   LRFCKKMSSDGKPIEPRVQFTNGGTCHPEHFDCVIRPRSLATEALVNSD-- 512
621A1 F. graminearum    LQFCKKVAQDGTPIEPRVQFTNGGTCHPEHFDCVIKPRNATATALINAS-- 511
621A2 N. haematococca   LEFRKKMGPDGSPIEPRVQFTNGGTCHPEHFECDIRPRSHKAAELIGANHD 516
621B1 A. clavatus       VSWRCPVDEAGREKRPEVQFSDGLSGVPDRFECEMQARDAESKEVL----- 498
                        : :   :   *   .*.*:*::* :                :
```
