# Supplementary material for: The Ultimate Micro-Exon: A Single Nucleotide Exon Is Required to Assemble Cytochrome P450 CYP621A Orthologs from Fusarium Species
Source: Int J Mol Sci. 2026 Feb 19;27(4):1979. doi: 10.3390/ijms27041979 (PMC12940212; doi:10.3390/ijms27041979)
Supplement: Supplementary file 1 [file ijms-27-01979-s001.zip › Figure S3.pdf]

Figure S3: Transcriptome evidence for CYP621A2 *Nectria haematococca/Fusarium solani*.

https://www.ncbi.nlm.nih.gov/sra/?term=fusarium+solani+SRX1810231

GSM2182753: FSONTC; Fusarium solani; RNA-Seq

strain FMR4391
tissue mycelium
protocol mycelium exposed to 48 hours to dimethyl-sulfoxide

```
SRX1810231 gnl|SRA|SRR3609449.10974918.1Length: 100

Query: 99   IPKGTVVYANAHAMAHDERIYRAPHDFNPDRYE 1
             IPKGTVVYANAHAMAHDERIYRAPHDFNPDRYE
Sbjct: 396  IPKGTVVYANAHAMAHDERIYRAPHDFNPDRYE 428

SRX1810231 gnl|SRA|SRR3609449.10974918.2Length: 100

Query: 2    LAPLGIPHKSLQDDVYQGMFIPKGTVVYANAHA 100
             LAPLGIPHKSLQDDVYQGMFIPKGTVVYANAHA
Sbjct: 376  LAPLGIPHKSLQDDVYQGMFIPKGTVVYANAHA 408

SRX1810231 gnl|SRA|SRR3609447.33844133.2Length: 100

Query: 3    SPLAPLGIPHKSLQDDVYQGMFIPKGTVVYAN 98
             SPLAPLGIPHKSLQDDVYQGMFIPKGTVVYAN
Sbjct: 374  SPLAPLGIPHKSLQDDVYQGMFIPKGTVVYAN 405

SRX1810231 gnl|SRA|SRR3609447.25291074.2Length: 100

Query: 14  WSPLAPLGIPHKSLQDDVYQGMFIPKGTV 100
            WSPLAPLGIPHKSLQDDVYQGMFIPKGTV
Sbjct: 373 WSPLAPLGIPHKSLQDDVYQGMFIPKGTV 401
```

11 reads from

strain FMR4391
tissue mycelium
protocol mycelium exposed to 48 hours to posaconazole

https://www.ncbi.nlm.nih.gov/sra/?term=fusarium+solani+SRX1810230

SRX1810230 gnl|SRA|SRR3609444.8194221.1Length: 100

GIPHKSLQDDVYQGMFIPKGTVVYANAHAMAHD

gnl|SRA|SRR3609444.8194221.2Length: 100

YIEHIVQEIYRWSPLAPLGIPHKSLQDDVYQGM

Note: just upstream of the PKG

gnl|SRA|SRR3609444.17343568.2Length: 100

IYRWSPLAPLGIPHKSLQDDVYQGMFIPKGTVV

SRX1810230 gnl|SRA|SRR3609445.7850576.1Length: 100

YRWSPLAPLGIPHKSLQDDVYQGMFIPKGTVVY

SRX1810230 gnl|SRA|SRR3609445.28658232.2Length: 100

QEIYRWSPLAPLGIPHKSLQDDVYQGMFIPKGT

gnl|SRA|SRR3609445.6764897.2Length: 100

EIYRWSPLAPLGIPHKSLQDDVYQGMFIPKGTV

gnl|SRA|SRR3609445.434239.2Length: 100

WSPLAPLGIPHKSLQDDVYQGMFIPKGTVVYAN

gnl|SRA|SRR3609445.434239.1Length: 100

QLDSVIGPDRLPNFSDRASLPYIEHIVQEIYRW

Note: 23 amino acids upstream of the PKG

gnl|SRA|SRR3609445.23302647.2Length: 100

IYRWSPLAPLGIPHKSLQDDVYQGMFIPKGTVV

gnl|SRA|SRR3609446.26312239.1Length: 100

IYRWSPLAPLGIPHKSLQDDVYQGMFIPKGTVV

gnl|SRA|SRR3609446.12254076.2Length: 100

IYRWSPLAPLGIPHKSLQDDVYQGMFIPKGTVV

strain FMR4391

tissue mycelium

protocol mycelium exposed to 48 hours to amphotericine

https://www.ncbi.nlm.nih.gov/sra/?term=fusarium+solani+SRX1810229

SRX1810229 gnl|SRA|SRR3609442.9370392.1Length: 100

PHKSLQDDVYQGMFIPKGTVVYANAHAMAHDE

gnl|SRA|SRR3609443.3469891.1Length: 100

IPKGTVVYANAHAMAHDERIYRAPHDFNPDRYE

gnl|SRA|SRR3609443.3469891.2Length: 100

PGLTLVTSPGIPHKSLQDDVYQGMFIPKGTVVY

gnl|SRA|SRR3609441.18495082.1Length: 100

SPGIPHKSLQDDVYQGMFIPKGTDHLQ-ELKQ has intron in place

gnl|SRA|SRR3609443.28803435.2Length: 100

PGIPHKSLQDDVYQGMFIPKGTDHLQ-ELKQFG has intron in place
